# QT interval prolongation related to psychoactive drug treatment: a comparison of monotherapy versus polytherapy

**DOI:** 10.1186/1744-859X-4-1

**Published:** 2005-01-25

**Authors:** Michela Sala, Alessandro Vicentini, Paolo Brambilla, Cristina Montomoli, Jigar RS Jogia, Eduardo Caverzasi, Alberto Bonzano, Marco Piccinelli, Francesco Barale, Gaetano M De Ferrari

**Affiliations:** 1 Department of Health Sciences-Section of Psychiatry, IRCCS Policlinico S. Matteo, University of Pavia, School of Medicine, Pavia, Italy; 2 Department of Cardiology, IRCCS Policlinico S. Matteo, University of Pavia, School of Medicine, Pavia, Italy; 3 Department of Health Sciences, University of Pavia, Pavia, Italy; 4 Department of Pathology and Experimental and Clinical Medicine, Section of Psychiatry, University of Udine School of Medicine, Udine, Italy; 5 Psychiatry Unit, Azienda Ospedaliera Universitaria Ospedale di Circolo e Fondazione Macchi di Varese, Presidio Ospedaliero del Verbano – Italy; 6 Section of Neurobiology of Psychosis, Institute of Psychiatry, London, UK

**Keywords:** antipsychotic, antidepressant, proarrhythmia, QTc interval

## Abstract

**Background:**

Several antipsychotic agents are known to prolong the QT interval in a dose dependent manner. Corrected QT interval (QTc) exceeding a threshold value of 450 ms may be associated with an increased risk of life threatening arrhythmias. Antipsychotic agents are often given in combination with other psychotropic drugs, such as antidepressants, that may also contribute to QT prolongation. This observational study compares the effects observed on QT interval between antipsychotic monotherapy and psychoactive polytherapy, which included an additional antidepressant or lithium treatment.

**Method:**

We examined two groups of hospitalized women with Schizophrenia, Bipolar Disorder and Schizoaffective Disorder in a naturalistic setting. Group 1 was composed of nineteen hospitalized women treated with antipsychotic monotherapy (either haloperidol, olanzapine, risperidone or clozapine) and Group 2 was composed of nineteen hospitalized women treated with an antipsychotic (either haloperidol, olanzapine, risperidone or quetiapine) with an additional antidepressant (citalopram, escitalopram, sertraline, paroxetine, fluvoxamine, mirtazapine, venlafaxine or clomipramine) or lithium. An Electrocardiogram (ECG) was carried out before the beginning of the treatment for both groups and at a second time after four days of therapy at full dosage, when blood was also drawn for determination of serum levels of the antipsychotic.

Statistical analysis included repeated measures ANOVA, Fisher Exact Test and Indipendent T Test.

**Results:**

Mean QTc intervals significantly increased in Group 2 (24 ± 21 ms) however this was not the case in Group 1 (-1 ± 30 ms) (Repeated measures ANOVA p < 0,01). Furthermore we found a significant difference in the number of patients who exceeded the threshold of borderline QTc interval value (450 ms) between the two groups, with seven patients in Group 2 (38%) compared to one patient in Group 1 (7%) (Fisher Exact Text, p < 0,05).

**Conclusions:**

No significant prolongation of the QT interval was found following monotherapy with an antipsychotic agent, while combination of these drugs with antidepressants caused a significant QT prolongation. Careful monitoring of the QT interval is suggested in patients taking a combined treatment of antipsychotic and antidepressant agents.

## Background

The QTc interval is a heart rate corrected value that measures the time between the onset and the end of electrical ventricular activity. Prolongation of this interval is considered a marker of the arrhythmogenic potential of a drug specifically linked to an increased risk of torsade de pointes ventricular tachycardia [1].

According to a document presented by the Committee for Proprietary Medicinal Products (CPMP) in 1997, normal subjects can be divided into three groups based on QTc interval length. For males, QTc values less than 430 ms are normal, between 431 and 450 ms are borderline and over 450 ms are prolonged. Whereas for females QTc values less than 450 ms are normal, between 451 and 470 ms are borderline and over 470 ms are prolonged [2].

This sex difference appears to be androgen driven and not determined by female hormones: at birth, QTc interval measurements are the same for male and female infants. At puberty, the male QTc interval shortens and remains shorter than its female counterpart by about 20 ms until ages 50 to 55 years, coincident with a decline in testosterone levels moreover, baseline QTc interval duration doesn't show significant fluctuations during the menstrual cycle and Hormone Replacement Therapy in postmenopausal age doesn't affect QTc interval [3].

In the above mentioned CPMP document it was also suggested that individual changes of QTc length of between 30 and 60 ms from baseline raises concern for the potential risk of drug induced arrhythmias [2].

Antipsychotics such as thioridazine, ziprasidone, quetiapine, risperidone, olanzapine or haloperidol have been suggested to prolong QTc interval [4-7].

Some authors reported that antidepressant drugs, including Selective Serotonine Reuptake Inhibitors (SSRI) (fluvoxamine, paroxetine and sertraline), Tricyclic Antidepressants (TCA) (amytriptiline, clomipramine, imipramine), and lithium can also prolong QTc interval [8-10].

Almost all drugs causing significant QT prolongation are known to interact with repolarizing potassium channels, particularly with the rapid component of delayed rectifier potassium currents (I_kr_), encoded by the human *Ether-a-go-go related *gene (HERG) [11].

However, TCA agents may affect the QTc interval primarily by their effect on sodium channels during depolarization [12]. Nonetheless TCA can also affect HERG potassium channels [13].

Drug trapping and structure-function studies suggest that the inner cavity of HERG channels is larger than other voltage-gated potassium channels and is therefore able to accommodate diverse chemical structures [14]. Among those drugs there are also SSRI like fluvoxamine [15], citalopram [16] and fluoxetine [17].

The combination of antipsychotic and antidepressant agents seems to have addictive effects on QTc interval [18].

We investigated the effects of polypharmacy on QTc in two groups of psychiatric female inpatients. Our null hypothesis was that patients treated with antipsychotics plus antidepressants or lithium would not have a greater QTc prolongation, if any, than patients treated with antipsychotics alone.

## Methods

A prospective naturalistic observational study was conducted in the Department of Psychiatry of San Matteo Hospital in Pavia. The study was approved by local Ethics Review Committee.

We chosed to recruit only women because of their higher risk of developing drug related arrythmias. Consecutive female inpatients admitted from August 2003 to April 2004, with schizophrenia, bipolar disorder or schizoaffective disorder, were considered eligible for the study. Diagnoses were made by two staff psychiatrists (one attending and one resident psychiatrist), after reaching a clinical consensus in accordance to the DSM IV.

Pharmacological and medical history were obtained.

Included patients had to be free from psychiatric medications for at least 48 hours. Patients who were taking fluoxetine untill three days before recruitment were excluded, because of the long half life of this drug (3–5 days). Also patients treated with depot preparations were excluded. Non psychoactive drugs, like cardiovascular drugs, were allowed only if they were not reported to alter QT interval.

Patients with disturbances of cardiac rate and rhythm, history of prolonged QTc, family history of sudden death, QTc interval greater than 470 ms in the ECG performed at admission, alterations of hepatic or renal function and substance abusers patients were also excluded.

For each subject, therapy was started according to the clinical evaluation of psychiatrist in charge of the patient. The first group (Group 1) included women who were treated with only an antipsychotic (concomitant benzotropine treatment was permitted, as also zolpidem for insomnia was), the second group (Group 2) was composed of female patients who started treatment with antipsychotics in association with either an antidepressant or lithium. The dosage equivalent of haloperidol was calculated [19].

Two ECGs were obtained for each patient, the first before the beginning of treatment and the second after four days of treatment with the patients on the full therapeutic daily dose of antipsychotic prescribed by the clinician, generally after one week from the beginning of the treatment, except for the patients treated with clozapine, who had the second ECG four days after the end of titration (generally after two weeks of therapy).

On the same day of the first ECG, blood samples were drawn for the evaluation of potassium serum levels from all the participants. When the second ECG was administered, blood was drawn to obtain potassium and additionally magnesium serum levels as well as serum levels of the antipsychotic agent.

Samples for plasmatic levels determination were drawn before the first drug dose in the morning. Serum antipsychotic levels were analyzed by high-performance liquid chromatography with ultraviolet detection.

The ECGs were obtained by standard 3-leads resting ECG procedure in the supine position and analyzed by a resident cardiologist (A. V.) who was blind to the patient's condition, study hypothesis, treatment status, serum levels of antipsychotics and was not involved in patient care. The QTc interval was calculated with the Bazzett formula. The QT interval was assessed in both DII and V2 leads. It was decided to focus on DII leads measurements due to the higher variability of measurements in precordial leads.

### Statistical Analysis

A repeated measures analysis of variance was used to test the effect of treatments on QTc (within subject factor: time of ECG examination, between subject factor: therapy group). Fisher Exact Test was used to compared the number of patients who exceed the threshold of borderline QTc values in Group 1 and Group 2. Indipendent T-Sample Tests were used to test the differences between baseline QTc values, ages and duration of illness using the statistical package Stata 7.0 (Stata Coorporation, 2001).

## Results

### Patients' characteristics

Our sample consisted of thirty eight women. Ninenteen were included in the Group 1 and ninenteen in the Group 2.

Age and duration of illness were comparable between the two groups (Table 1).

**Table 1:** Diagnosis, duration of illness, psychoactive treatment before recruitment and age of patients

	Diagnosis	Comorbid Disorders (N)	Duration of illness (yr)	Psychoactive treatment 48 hours before recruitment (N)	Age (yr) Mean ± SD	Age (yr) Range
Group 1 Patients in monotherapy	Schiz 17 Schizoaf 2	0	5,8 ± 5	Haloperidol 3 Risperidone 2	45,7 ± 15	22–77
Group 2 Patients in politherapy	Schizoaf 10 Bip Dis 3 Schiz 6	An Nervosa 2 Alc Abuse 2	4,2 ± 3	Venlafaxine 2 Haloperidol 1 Risperidone 2 Atenolole 2 Lacipidine 1 Amlodipine 1 Ranitidine 1	45,79 ± 12,8	26–74

Schizoaf: Schizoaffective Disorder; Schiz: Schizophrenia; Bip Dis: Bipolar Disorder; An Nervosa: norexia Nervosa; Alc Abuse: Alcohol Abuse yr: years

Data on diagnosis and previous pharmacological treatment of patients are reported in Table 1.

Among the nineteen patients in Group 1, five were treated with haloperidol, five with olanzapine, five with risperidone and four with clozapine; among the nineteen patients of group 2, five started haloperidol, eight olanzapine, four risperidone and two quetiapine. Antidepressant used were escitalopram (two patients), citalopram (three patients), mirtazapine (four patients), paroxetine (one patients), sertraline (two), fluvoxamine (one patient), venlafaxine (three patients), clomipramine (two patients). Three patients started also lithium treatment.

The mean antipsychotic doses, equivalent doses and mean plasmatic levels are reported in Table 2 (See Additional file 1 ). Potassium and magnesium serum levels were always within the normal range for all subjects.

### QTc interval

Mean baseline QTc intervals were similar in the two groups: 422 ± 26 ms in group 1 and 414 ± 22 ms in group 2 (Indipendent T-test p > 0,5). One patient in the monotherapy group, who started clozapine treatment, was excluded from this analysis and from pre-post treatment comparisons because QTc in DII was not measurable with sufficient accuracy.

Three patients in the first group had QTc values that exceeded 450 ms at baseline while no patients in Group 2 exceeded this threshold before starting treatment.

**Figure 1:**
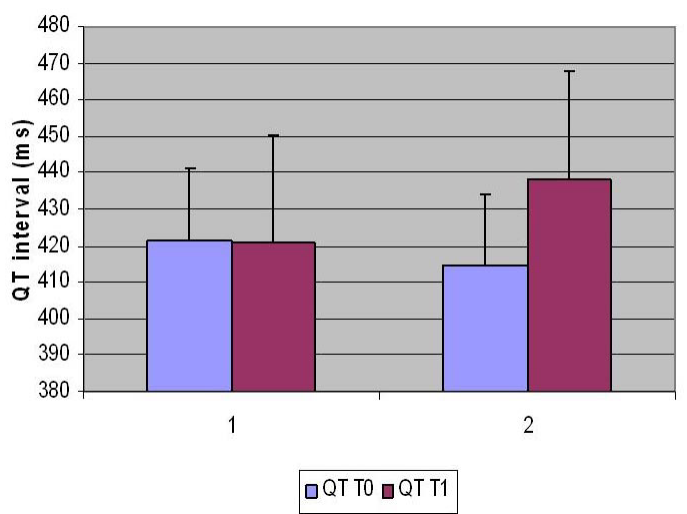
Mean QTc (bars indicate standard deviations) values at baseline (T0) and after four days at full dosage (T1) of antipsychotic therapy, in the monotherapy (1) and politherapy (2) groups.

The average QTc interval after treatment was 421 ± 20 ms in the monotherapy group (range 391–452) and 438 ± 30 and in the polytherapy group (range 379–488) (Indipendent T test, p < 0,05) (see figure 1).

Compared with the baseline, mean QTc change after treatment was – 1 ± 30 ms in Group 1 and 24 ± 21 ms in Group 2 (repeated measures ANOVA p < 0,05).

After treatment only one patient in Group 1 reached the threshold for borderline values of QTc interval in comparison to seven patients in Group 2 (Fisher Exact Text p < 0,05).

Moreover, in Group 2 two patients had a QTc exceeding 480 ms.

The highest prolongations of QTc intervals (66 ms and 55 ms) were found in two patients taking risperidone, the first in association to clomipramine and the second in association to escitalopram. However in these two cases plasmatic dosages of antipsychotics were not higher than in other patients who reported a shorter QTc prolongation.

## Discussion

We found that the psychiatric population treated with antipsychotic monotherapy had much less risk of developing an increase in QTc interval compared to those treated with antipsychotics plus an antidepressant or lithium.

Two main mechanisms seem to operate in determining the prolongation of QTc interval during treatment with different combinations of psychoactive drugs. The first is the synergic blockade of the HERG potassium channels, the second is the increase in drug levels (with subsequent augmented risk of cardiotoxicity) due to metabolic interactions between drugs that share the same metabolic pathway [20]. This mechanism may be particularly relevant in subject with genetic-determined impairment of CYP2D6 and CYP3A4 drug-metabolizing enzymes (poor metabolizer subjects) [21].

In our study, metabolic interactions leading to abnormal elevation of serum levels of antipsychotics did not seem to be the principal determinant of the greater QTc prolongation in the group with combined therapy. Indeed serum levels of antipsychotics were all within or under the expected range after therapeutic dosing in both groups, they were not higher in Group 2 compared to Group 1 and the highest prolongations observed were not associated with the highest antipsychotic serum levels.

Recently, Harringan et al [22] analyzed, in a prospective randomized study, the effects of six antipsychotics on the QTc interval; they found that each of the antipsychotics were associated with measurable QTc prolongation which was not augmented by concomitant use of metabolic inhibitors, even if in their study plasmatic levels of antipsychotics raised after the addition of the specific metabolic inhibitor.

In our study, the combination of different drugs doesn't seem to cause strong interactions on drug metabolism. However, in our sample, the combination of drugs that specifically interfere in their own methabolism, like fluvoxamine and olanzapine, paroxetine and risperidone, were avoided by clinicians.

Antidepressant used in our study have a mild inhibitory action on antipsychotic methabolism and this can explain why antipsychotic serum levels didn't raise in Group 2 compared to Group 1. It is reassuring to find that significant pharmacokinetic interactions do not occur when the antipsychotics studied were coadministered with antidepressant commonly used in clinical practice.

If the metabolic interactions do not seem to be the most important explanation for our results, an alternative explanation might be the synergic actions of different drugs on ion channels.

The two patients with the highest prolongations were both taking risperidone, which is noted to block the I_kr _current [23,24]. Actually, many psychotropic drugs share this capacity to inhibit I_kr_current, including not only antipsychotic agents but also antidepressant agents like citalopram, fluoxetine, paroxetine [16,23]. Those agents may have synergic effect when used in combination.

This study has several limitations, most of them related to the naturalistic setting of this study: we chosed to administer the second ECG after four days of therapy at full dosage (generally after one week from recruitment) because all antipsychotic used reached the steady-state in 3–5 days. Actually we couldn't chose a longer interval between the first and the second ECG because the average duration of recovery in our ward is 8,5 days. Dosing was clinically determined for symptom response by the treating psychiatrist and hence, doses varied within and between groups. Moreover statistical comparison of QTc interval changes among agents was not possible because of the small number of the samples.

Finally we didn't measure antidepressant serum levels. Consistently with data reported in literature, we thought that antipsychotic would have been the principal drugs involved in QTc prolongation, while antidepressant would have only a role of potentiating agents. Actually, serum levels of antidepressants would have helped to explain the greater prolongation observed in Group 2.

## Conclusions

Prolongation of the QT interval by a non cardiovascular drug including notably an antipsychotic agent is considered a good marker of the arrythmogenic potential of that agent [1].

Psychiatric patients had been identified as a population at risk for cardiovascular problems [12,25]. Mortality rates are higher in psychiatric patients than in the general population [26] and the pharmacological treatment itself might produce side effects that affect mortality from causes other than suicide [27].

There is a vast amount of evidence available showing the effect of a single antipsychotic on QTc interval however there is not much evidence obtained for clinical populations treated with a different combinations of drugs.

Interestingly, the response to repolarization prolonging stimuli is "patient-specific" [9]. Thus, detecting the patients with a reduced repolarization reserve [28,29] will lead to personalized psychotropic therapy according to the predisposition of that patient to develop cardiac side effects with a certain drug.

In view of the fact that psychiatric patients are considered high risk subjects and since they frequently show electrolytes unbalances [30], an accurate monitoring of the QTc interval before and after the beginning of treatment appears warranted, particularly for patients taking multiple psychoactive drugs, sharing QTc prolonging properties.

Further observational studies on larger samples of patients, comparing QTc intervals, plasmatic levels of antipsychotics and daily doses of psychotropic drugs are necessary to perform statistical comparisons for each kind of antipsychotic and for each kind of antipsychotic-antidepressant association commonly used in clinical practice.

## Competing interest

The author(s) declare that they have no competing interests.

## Author's contributions

MS recruited and assessed participants and conceived of the study, and participated in its design and coordination. AV and GMD read ECGs and measured the QTc and analysed the results. CM ran the statistical analysis. AB and EC participated in the assessment of participants. MP, PB, JRSJ and FB participated in its design and coordination and the interpretation of results.

## Supplementary Material

Additional File 1 Additional file 1: Table 2.doc Click here for file
