# Supplementary material for: QT interval prolongation related to psychoactive drug treatment: a comparison of monotherapy versus polytherapy
Source: Ann Gen Psychiatry. 2005 Jan 25;4:1. doi: 10.1186/1744-859X-4-1 (PMC1088007; doi:10.1186/1744-859X-4-1)
Supplement: Additional File 1 — Table 2.doc [file 1744-859X-4-1-S1.doc]

Table 2. **Mean oral daily dose , plasmatic levels and equivalent dose of antipsychotics in the group of patients receiving monotherapy and in the group receiving polytherapy**

|  |  |  | Patients with monotherapy  **Group 1** | | | | **Patients with polytherapy**  **Group 2** | | | | | |
| --- | --- | --- | --- | --- | --- | --- | --- | --- | --- | --- | --- | --- |
| **antips** | **antips CYP 450 pathway** | **Therap serum levels (ng/ml)** | n. pts | mean dose (mg/die) | mean serum level (ng/ml) | Mean  hal equivalent dose (mg/die) | n. pts | mean dose (mg/die) | mean serum level (ng/ml) | mean  hal equivalent dose (mg/die) | antidep used  (n. pts) | antidep CYP 450 pathway |
| **hal** | **CYP3A4 CYP2D6** | **5-15 ng/ml** | 5 | 4,8 ± 2 | 3,9 ± 2 | 4,8 | 5 | 4±1 | 3,54± 1 | 3 | Cit 2  Mir 1  Lit 1  Ser1 | **CYP2D6**  **CYP3A4**  **CYP1A2** |
| **ola** | **CYP1A2** | **10-100 ng/ml** | 5 | 14 ± 5 | 38,4 ± 16 | 10,5 | 8 | 9±5 | 41,8 ± 28 | 6,5 | Ven 3  Mir 2  Par 1  Ser 1  Cit 1 | **CYP2D6**  **CYP3A4**  **CYP1A2** |
| **risp** | **CYP2D6** | **6- 70 ng/ml**  **(ris+9Ohrisp)** | 5 | 3 ± 1 | 29,4 ± 21 | 6 | 4 | 3 ± 2 | 26,3 ± 12 | 6,8 | Clom 2  Escit 1  Fluv 1 | **CYP2D6**  **CYP3A4**  **CYP1A2** |
| **clo** | **CYP1A2**  **CYP3A4** | **200-1200 ng/ml**  **(clo+Nor clo)** | 4 | 317 ± 144 | 593 ± 243 | 9 | - | - | - | - | - |  |
| **que** | **CYP3A4** | **140-365 ng/ml** | - | - | - | - | 2 | 400 ± 283 | 55 ± 21 | 10,7 | Esc+Lit 1  Mir+Lit 1 | **CYP2D6**  **CYP3A4**  **CYP1A2** |

Hal : haloperidole, ola: olanzapine; clo: clozapine; Nor clo: nor-clozapine; que: quetiapine; risp: risperidone; 9OH risp: idrossiriseridone; mir: mirtazapine; cit: citalopram; esc:escitalopram; ser:sertraline; ven:venlafaxine; clom: clomipramine; par: paroxetine; lit: lithium; pts: patients; antid: antidepressant; antips: antipsychotic.
